# Large Resectable Pancreatic Cancer is Associated with Futile Surgery: A Resectable But Not Curable Disease?

**DOI:** 10.1245/s10434-025-18511-2

**Published:** 2025-10-27

**Authors:** Sho Kiritani, Yoshikuni Kawaguchi, Yusuke Kazami, Satoru Abe, Yujiro Nishioka, Yuichiro Mihara, Akihiko Ichida, Takeshi Takamoto, Nobuhisa Akamatsu, Kiyoshi Hasegawa

**Affiliations:** Hepato-Biliary-Pancreatic Surgery Division, Department of Surgery, Graduate School of Medicine, The University of Tokyo, Tokyo, Japan

**Keywords:** Resectable pancreatic cancer, Futile surgery, Pancreatectomy, Tumor diameter, Local recurrence

## Abstract

**Background:**

A subset of patients with resectable pancreatic cancer (PC) experience early recurrence after curative-intent pancreatectomy and subsequently face a poor prognosis. However, the underlying factors associated with such a futility of surgery remain unclear.

**Methods:**

This retrospective cohort study analyzed 369 patients with resectable PC who underwent curative pancreatectomy between 2008 and 2022. Futile surgery was defined as recurrence within 8 months postoperatively. Receiver operating characteristic curve and Youden’s index distribution were used to identify the optimal tumor diameter cutoff, which then was applied to survival analysis.

**Results:**

A diameter of 3 cm corresponded to the peak of Youden’s index and was adopted as the cutoff value. Patients were classifed into the large group (≥ 3 cm; *n* = 206) and small group (< 3 cm; *n* = 163). The median overall survival (OS) was significantly shorter in the large group (2.3 years) than in the small group (7.6 years) (*p* < 0.01). The large group with neoadjuvant therapy had a median OS of 3.4 years, comparable with 2.3 years for the large group with upfront surgery (*p* = 0.33). Multivariable analysis identified the large group as a poor independent prognostic factor (hazard ratio, 2.2; *p* < 0.01). Even after R0 resection, local recurrence was more frequently observed in the large group (34.1% vs 11.8%; *p* < 0.01).

**Conclusions:**

A tumor size of 3 cm was associated with surgical futility in resectable pancreatic cancer and served as a useful factor for fine stratification of long-term postoperative outcomes.

**Supplementary Information:**

The online version contains supplementary material available at 10.1245/s10434-025-18511-2.

Pancreatic cancer (PC) remains one of the most lethal malignancies, with a poor prognosis and an increasing impact on cancer-related mortality worldwide. Although surgical resection remains the only curable treatment option, unresectable disease is diagnosed for more than 80% of patients.^1,2^

Surgical resection remains the only potential treatment option for pancreatic cancer, and patients with resectable PC have a significant chance of survival from surgery compared with those who have unresectable disease. In recent years, the concept of resectable PC, borderline PC, and unresectable PC has gained widespread recognition. However, this is an anatomic classification, not an oncologic one.^3^ Among patients with PC classified as resectable, a subset is known to experience early postoperative recurrence, which is associated with a poor prognosis and serves as a representative example of surgical futility.^4,5^ Identifying the factors associated with futile surgery is of critical importance for the resectable PC population.

Tumor diameter is a definitive prognostic indicator that corresponds with the T factor in the tumor-node-metastasis (TNM) classification.^6,7^ However, the optimal cutoff value for predicting futile surgery in resectable PC remains unknown, and the detailed postoperative course of this population is unclear. This study aimed to assess the association between tumor diameter and futile surgery for resectable PC and postoperative survival outcomes.

## Methods

### Study Populations

This retrospective cohort study was approved by the Institutional Review Board of The University of Tokyo Hospital (Approval No. 2158-11). The clinical data of patients with histologically diagnosed pancreatic cancer who underwent curative-intent pancreatectomy at our hospital between January 2008 and December 2022 were retrieved from our prospectively maintained database. For patients who received preoperative treatment, tumor marker values before preoperative treatment were used. To focus exclusively on resectable PC, the study excluded pancreatic resections for borderline PC or conversion surgeries for unresectable PC, as defined by the National Comprehensive Cancer Network guidelines.^3^

### Perioperative Treatment Strategy

To assess resectability and determine TNM staging as defined by the eighth edition of the Union for International Cancer Control (UICC), dynamic contrast-enhanced computed tomography (CT) and tumor markers (carcinoembryonic antigen and carbohydrate antigen 19-9 [CA19-9]) were evaluated for all the patients. Endoscopic ultrasonography (EUS) and magnetic resonance imaging (MRI) were performed if required.

Our treatment strategy for resectable PC shifted from upfront surgery to neoadjuvant chemotherapy (two courses of gemcitabine and S-1 [GS]) in 2019.^8^ As an oral anticancer agent, S-1 was developed to enhance the efficacy and reduce the side effects of fluorouracil, combining three components: tegafur, gimeracil, and oteracil. The patients with markedly elevated CA19-9 levels (> 500 U/mL) received four courses of gemcitabine/nab-paclitaxel therapy.^9^

We performed pancreaticoduodenectomy with regional lymph node dissection for pancreatic head cancer and distal pancreatectomy for pancreatic tail cancer. For pancreatic body cancer, we chose pancreaticoduodenectomy, distal pancreatectomy, or total pancreatectomy, depending on the approach that ensured R0 resection. Combined resection and reconstruction were performed for cancers involving the superior mesenteric and portal veins. The nerve plexus of the superior mesenteric artery was generally preserved. However, en bloc resection of the nerve plexus was performed in patients with suspected tumor invasion.

A gemcitabine-based regimen was used as adjuvant chemotherapy until 2012, and oral S-1 was administered from 2013 onward.^10,11^ Both regimens were generally administered for a duration of 6 months.^12^

### Pathologic Assessment

The pathologic diagnosis was determined by experts in pancreatic tumor pathology. Pathologic data, including tumor diameter, tumor differentiation, microscopic lymphatic invasion, vascular invasion, and neural invasion, were collected. We also evaluated whether the tumors extended beyond the pancreas. With regard to surgical curability, R0 resection was defined as having a histologically negative margin, whereas R1 resection was defined as having a positive margin (the 0-mm rule).^13^ Margin status was separately assessed for the pancreatic cut margin, bile duct cut margin, and dissected peripancreatic margin. The number of resected lymph nodes and metastases were counted. The eighth edition of the Union for International Cancer Control (UICC) staging system was used to determine the TNM stage.^7^

### Follow-Up Evaluation

Postoperatively, tumor markers were checked every 3 months, and computed tomography (CT) was performed at the time of completion of adjuvant chemotherapy and every 6 months thereafter to monitor for recurrence. Fluorodeoxyglucose-positron emission tomography or histologic examination was performed as required. Local recurrence was defined as recurrence in the surgical bed such as in the soft tissue along the celiac or superior mesenteric artery or aorta, or around the pancreaticojejunostomy site.

In cases of recurrence, chemotherapy is the first line of treatment. For metachronous pancreatic cancer occurring in the remnant pancreas, complete pancreatectomy was considered if the tumor was locally resectable and no distant metastases were detected.^14^

### Endpoint Setting and Definition of Futile Surgery

Postoperative long-term outcomes were evaluated using disease-specific survival (DSS) and recurrence-free survival (RFS). The DSS period was calculated as the time from the day of surgery to cancer-related death and was censored at the date of the last follow-up assessment for the patients who were alive or had died of other causes. The RFS period was calculated as the time from the day of surgery to the day of recurrence identification by imaging and/or histologic examination or death and was censored at the date of the last follow-up assessment for the patients who were alive without any recurrence. Futile surgery was defined as recurrence happening within 8 months postoperatively because such recurrence would be diagnosed during the course of multimodal treatment (i.e.,, surgery followed by 6 months of adjuvant chemotherapy initiated within 2 months postoperatively).

### Statistical Analysis

Continuous and categorical variables are expressed as medians, ranges, and frequencies with percentages. The follow-up period was evaluated in the censored cases. The Mann–Whitney *U* or chi-square test was used to compare the two groups. Tumor diameter was defined based on pathologic evaluation. Predictive performance of tumor diameter for futile surgery was evaluated using receiver operating characteristic (ROC) curves. The Youden’s index (sensitivity + specificity − 1) was calculated at each threshold, and Youden’s index distribution was plotted to identify the threshold yielding the maximum Youden’s index, which was considered the optimal cutoff value.

We determined the cutoff values for the continuous variables, CA19-9 and the number of positive lymph node metastases using Youden's index, and included them in the multivariable analysis. The Kaplan–Meier method and the log-rank test were used to estimate the survival rate and compare the survival curves between the two groups. Variables with *p* value lower than 0.05 were applied to the subsequent multivariable logistic analysis and Cox proportional hazards model analysis using the backward stepwise method. A *p* value lower than 0.05 was defined as statistically significant. All statistical analyses were performed using IBM SPSS Statistics software (version 29.0; IBM Japan Ltd., Tokyo, Japan) and SAS, version 9.4 (SAS Institute, Inc.; Cary, NC, USA).

## Results

### Study Population

During the study period, curative-intent pancreatectomy for pancreatic cancer was performed for 458 patients. After 80 borderline resectable and 9 conversion surgeries for unresectable tumors were excluded, 369 patients were left for analysis. For 83 (22.5%) of these patients, futile surgery was performed.

**Fig. 1:**
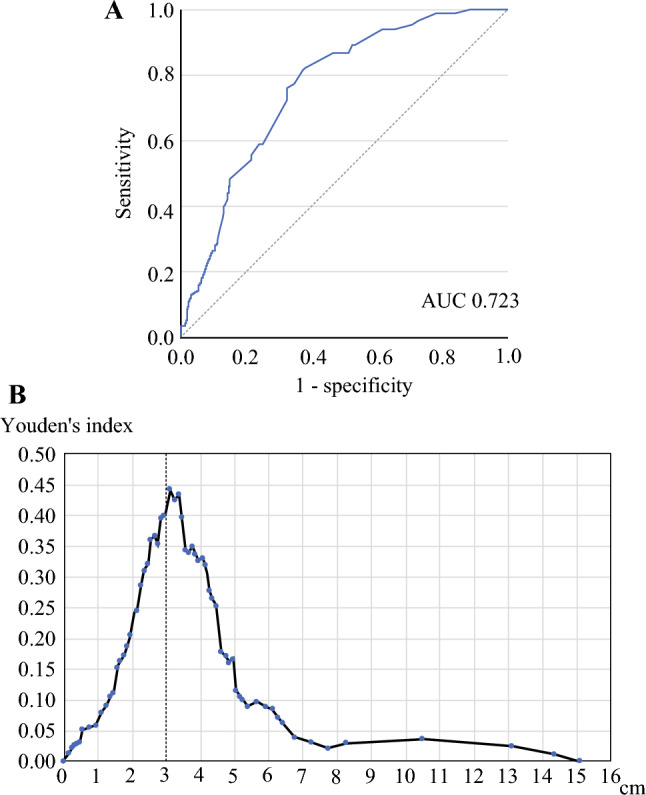
Receiver operating characteristics (ROC) curve and Youden’s index plot illustrating the relationship between futile surgery and tumor size. **A** The ROC curve shows an area under the curve (AUC) of 0.723. **B** The Youden’s index plot: the x-axis represents tumor diameter, and the y-axis represents the Youden’s index (sensitivity + specificity − 1)

Figure 1A illustrates the relationship between futile surgery and tumor diameter. Figure 1B shows a plot of the Youden’s index for each tumor diameter. Based on these findings, a tumor diameter of 3 cm was determined to be the closest to the maximum Youden’s index and was considered the optimal cutoff point. The futile-surgery group had a median overall survival (OS) of only 1.2 years (5-year DSS rate, 0%), which was significantly shorter than for the group with no futile surgery (median OS, 6.7 years; 5-year DSS rate, 54.3%; *p* < 0.01; Fig. S1).

### Backgrounds and Surgical Information

Because a tumor diameter cutoff of 3 cm may be better to dichotomize a risk for futile surgery for patients with resectable PC (Fig. 1), 369 patients were divided into 206 patients with a pancreatic cancer size of ≥ 3 cm (large group) and 163 patients with a pancreatic cancer size of < 3 cm (small group). A comparison of the background and surgical information between the large and small groups is presented in Table 1. In the large group, tumor location was more frequently in the pancreatic head compared with the small group (64.6% vs 18.5%; *p* < 0.01). The median CA19-9 level was higher in the large group (115 vs 52 U/mL; *p* < 0.01). The rates of neoadjuvant or adjuvant chemotherapy did not differ significantly between the groups (17.5% vs 20.2% [*p* = 0.50], 73.3% vs 71.2% [*p* = 0.65]). The completion rates for neoadjuvant and adjuvant chemotherapy were respectively 77.8% and 55.0% (*p* = 0.27) in the large group versus 87.9% and 79.3% in the small group (*p* = 0.01). Because of the difference in tumor location, surgical procedures also differed significantly, with a higher rate of pancreaticoduodenectomy performed in the large group (60.2% vs 48.5%; *p* < 0.01).

**Table 1:** Comparison of theackgrounds and surgical information between the large and small groups

Variables	Large group	Small group	*p* Value^a^
	(*n* = 206) *n* (%)	(*n* = 163) *n* (%)	
Backgrounds			
Median age: years (range)	72 (20–88)	71 (40–89)	0.53
Male sex	134 (65.0)	103 (63.2)	0.71
Tumor location, head/body-tail	133/73	79/84	< 0.01
Median BMI: kg/m^2^ (range)	21.9 (12.9–36.5)	22.2 (15.4–34.6)	0.22
Median CEA: ng/mL (range)	3.9 (0.6–36.7)	3.7 (0.8–114.2)	0.32
Median CA19-9: U/mL (range)	115 (1–13310)	52 (1–3135)	< 0.01
Median DUPAN-2: U/mL (range)	166 (14–16000)	45 (19–7390)	< 0.01
Median span-1: U/mL (range)	48 (10–1998)	24 (10–972)	< 0.01
Neoadjuvant chemotherapy, yes	36 (17.5)	33 (20.2)	0.50
Completion chemotherapy	28 (77.8)	27 (87.9)	0.27
Adjuvant chemotherapy, yes	151 (73.3)	116 (71.2)	0.65
Gemcitabine-based	47 (31.1)	29 (25.0)	
S1 administration	103 (68.2)	87 (75.0)	
Others	1 (0.7)	0 (0.0)	
Completion chemotherapy	83 (55.0)	92 (79.3)	0.01
*Surgical information*			
Surgical procedure			< 0.01
Pancreaticoduodenectomy	124 (60.2)	79 (48.5)	
Distal pancreatectomy	74 (35.9)	82 (50.3)	
Total pancreatectomy	8 (3.9)	1 (0.6)	
Other limited pancreatectomy	0 (0.0)	1 (0.6)	
Median operative time: min (range)	511 (163–951)	414 (140–1148)	< 0.01
Median blood loss: mL (range)	595 (40–4010)	390 (10–4350)	< 0.01
Blood transfusion	31 (15.0)	8 (4.9)	< 0.01
Portal resection	57 (27.7)	25 (15.3)	< 0.01
Clavien–Dindo grade > 3a	44 (21.4)	50 (30.7)	0.04
Postoperative hospital stay: days (range)	22 (7–151)	21 (7–105)	0.45

BMI, body mass index; CEA, carcinoembryonic antigen; CA19-9, carbohydrate antigen 19-9; DUPAN-2, Duke pancreatic monoclonal antigen type 2; Span-1, s-pancreas antigen-1

^a^*p* Values were calculated using the Mann–Whitney U test or chi-square test, as appropriate

### Pathologic Findings

A comparison of the pathologic findings between the large and small groups is presented in Table S1. The histologic type was adenocarcinoma in 347 cases, whereas in the remaining cases, it was categorized as “others”. These included adenosquamous carcinoma (*n* = 14), mucinous carcinoma (*n* = 3), acinar cell carcinoma (*n* = 3), and anaplastic carcinoma (*n* = 2).

Tumor differentiation did not differ significantly between the two groups (*p* = 0.12). However, the large group more frequently exhibited microscopic lymphatic invasion (64.1% vs 35.6%; *p* < 0.01), venous invasion (93.2% vs 80.4%; *p* < 0.01), and perineural invasion (94.7% vs 73.0%; *p* < 0.01). The large group also had significantly higher rates of extrapancreatic invasion (95.6% vs 71.8%; *p* < 0.01) and lymph node metastasis (70.9% vs 43.6%; *p* < 0.01). Additionally, the R1 resection rate was higher in the large group (20.4% vs 11.7%; *p* = 0.03).

### Factors Associated With Futile Surgery and Validation of the Predictive Model

Table 2 shows the results of multivariable logistic regression analysis of factors associated with early recurrence and futility of surgery. Similar to tumor diameter, the cutoff values for CA19-9 and the number of positive lymph node metastases were determined using Youden's index, resulting in 188 U/mL for CA19-9 and two nodes as the optimal threshold. The independent factors identified as associated with futility of surgery were a diameter of ≥ 3 cm (odds ratio [OR], 5.74; *p* < 0.01) and venous invasion positivity (OR, 4.22; *p* = 0.04).

**Table 2:** Logistic regression analysis of factors associated with futility of surgery

Variables	*n*	Univariable			Multivariable		
		OR	95% CI	*p* Value	OR	95% CI	*p* Value
Age ≥ 75 years	140	1.10	0.67–1.83	0.70			
Tumor location, head	212	1.73	1.03–2.89	0.04	1.25	0.70–2.21	0.45
Neoadjuvant chemotherapy, no	300	0.95	0.51–1.77	0.88			
Tumor diameter ≥ 3 cm	206	7.43	3.78–14.59	< 0.01	5.74	2.80–11.81	< 0.01
CA19-9 ≥ 188 U/mL	106	1.93	1.15–3.22	0.02	1.28	0.73–2.24	0.38
Tumor differentiation, other than well-differentiated	200	1.47	0.89–2.41	0.13			
Lymphatic invasion, yes	190	3.13	1.84–5.34	< 0.01	1.65	0.86–3.16	0.13
Venous invasion, yes	323	7.36	1.75–31.06	0.01	4.22	1.12–21.13	0.04
Perineural invasion, yes	314	3.31	1.27–8.58	0.01	0.67	0.21–2.14	0.50
Positive lymph nodes metastasis, ≥ 2	159	2.63	1.59–4.34	< 0.01	1.22	0.66–2.24	0.53
R1 resection, yes	61	1.42	0.76–2.64	0.27			
Adjuvant chemotherapy, no	102	1.57	0.93–2.65	0.09			

OR, odds ratio; CI, confidence interval; CA19-9, carbohydrate antigen 19-9

Validation of early recurrence using the 3-cm tumor size cutoff was performed for 63 patients with resectable pancreatic cancer who underwent curative pancreatectomy between January 2023 and June 2024. The ROC curve had a sensitivity of 75.0%, a specificity of 62.8%, and an AUC of 0.691. Compared with the 369 patients in the main cohort of this study, for whom the sensitivity was 86.7% and the specificity was 53.1%, the sensitivity was slightly lower, whereas the specificity was slightly higher, and the AUC was nearly equivalent.

### Long-Term Survival

**Fig. 2:**
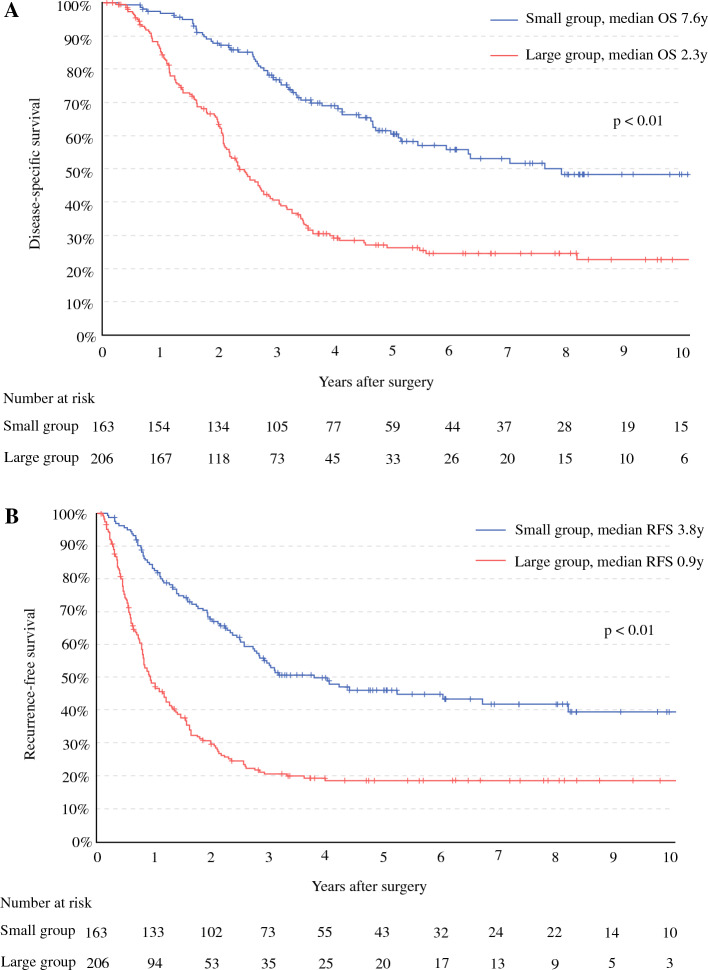
**A** Comparison of Kaplan–Meier curves for disease-specific survival between the small and large groups. The median overall survival (OS) was 7.6 years in the small group and 2.3 years in the large group, with a log-rank test showing a *p* value lower than 0.01. **B** Comparison of recurrence-free survival (RFS) between the small and large groups. The median RFS was 3.8 years in the small group and 0.9 years in the large group (*p* < 0.01)

The Kaplan–Meier curve comparing DSS between the large and small groups is presented in Fig. 2A. The median follow-up period was 4.3 years. The median OS for the large group was 2.3 years, which was significantly shorter than the 7.6 years for the small group (*p* < 0.01). The Kaplan–Meier curve comparing the RFS is shown in Fig. 2B. The median follow-up period was 4.7 years. The median recurrence-free time for the large group was 0.9 years, significantly shorter than the 3.8 years for the small group (*p* < 0.01).

**Fig. 3:**
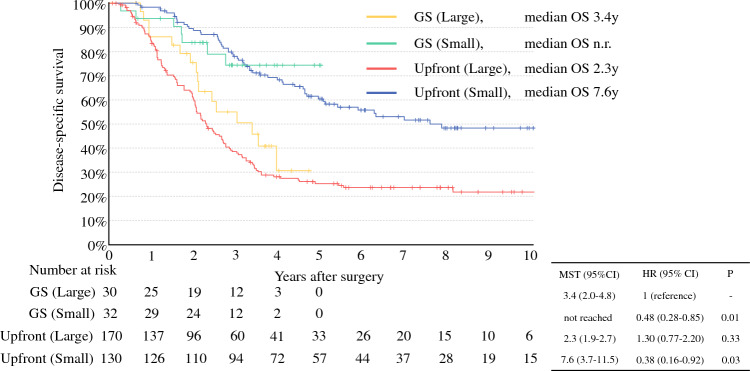
Kaplan–Meier curves stratified by use of GS therapy in the large and small groups. The GS (large) group had a significantly worse prognosis than the GS (small) group (*p* = 0.01) or the upfront (small) group (*p* = 0.03) and showed no significant difference in prognosis compared with the upfront (large) group (*p* = 0.33)

Figure 3 shows a comparison of the Kaplan–Meier curves by dividing the large and small groups each into four subgroups based on whether they underwent neoadjuvant GS therapy or upfront surgery. This comparison was performed to evaluate the efficacy of preoperative GS therapy in the large group. The median OS of the GS (large) group was 3.4 years, significantly shorter than that of the GS (small) group (median OS, not reached; *p* = 0.01) and upfront (small) group (median OS, 7.6 years; *p* = 0.03). However, compared with the upfront (large) group (median OS, 2.3 years; *p* = 0.33), there was no significant difference.

In the Cox proportional hazard model with disease-specific death as the event (Table S2), univariable analysis identified the following as poor prognostic factors: large group (hazard ratio [HR]: 2.65, *p* < 0.01), CA19-9 ≥ 500 U/mL (HR, 2.22; *p* < 0.01), differentiation other than well-differentiated (HR, 1.41; *p* = 0.02), lymphatic invasion (HR, 2.41; *p* < 0.01), venous invasion (HR, 2.40; *p* < 0.01), perineural invasion (HR, 2.68; *p* < 0.01), lymph node metastasis (HR, 2.08; *p* < 0.01), and R1 resection (HR, 2.04; *p* < 0.01).

In the multivariable analysis, the independent poor prognostic factors were identified as large group (HR, 2.15; *p* < 0.01), CA19-9 ≥ 500 U/mL (HR, 1.54; *p* = 0.02), lymphatic invasion (HR, 2.01; *p* < 0.01), and R1 resection (HR, 1.84; *p* < 0.01). It is particularly interesting that tumor size was a stronger predictor of DSS than well-established prognostic factors such as lymph node metastasis, CA19-9 levels, and R1 resection.

### Recurrence Pattern

A comparison of the recurrence patterns between the large and small groups is presented in Table 3. During the observation period, the number of recurrences was 158 (76.7%) in the large group and 82 (50.3%) in the small group. Local recurrence, liver metastasis, and peritoneal dissemination were more frequent in the large group (all *p* < 0.01), whereas the rate of recurrence in the residual pancreas was higher in the small group (*p* < 0.01).

**Table 3:** Comparison of postoperative recurrence patterns between the large and small groups^a^

Variables	Large group	Small group	*p* Value
	(*n* = 158) *n* (%)	(*n* = 82) *n* (%)	
Local recurrence	73 (35.4)	25 (15.3)	< 0.01
Liver metastasis	61 (29.6)	19 (11.7)	< 0.01
Peritoneal dissemination	35 (17.0)	12 (7.4)	< 0.01
Lung metastasis	27 (13.1)	14 (8.6)	0.14
Residual pancreas	7 (3.4)	19 (11.7)	< 0.01
Others	11 (5.3)	4 (2.5)	0.16

^a^Chi-square test was used; duplicate experiments also were performed

**Fig. 4:**
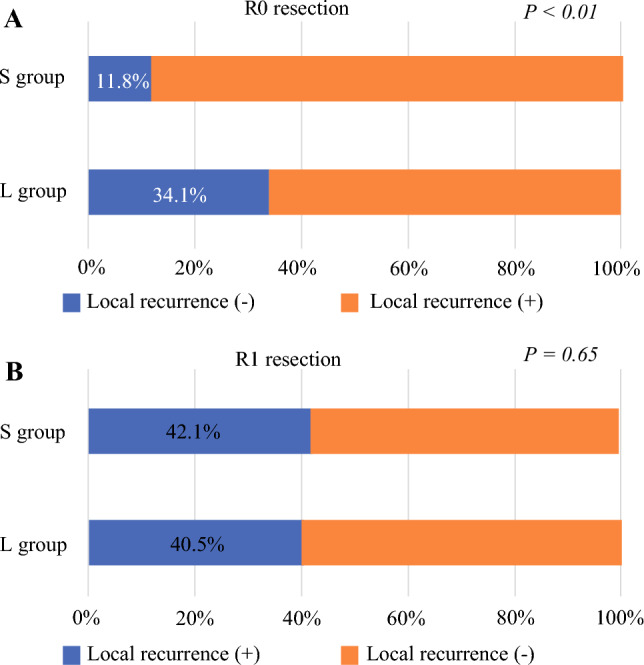
**A** The local recurrence rate was significantly higher in the large group (34.1%) than in the small group (11.8%) (*p* < 0.01), even with R0 resection. **B** The two groups did not differ significantly in R1 resection (42.1% vs 40.5%; *p* = 0.65).

A comparison of local recurrence rates between the large and small groups is presented with stratification of R status in Fig. 4. The local recurrence rate in cases of R1 resection did not differ significantly between the large and small groups (40.5% vs 42.1%; *p* = 0.65). However, in cases of R0 resection, the local recurrence rate was significantly higher in the large group than in the small group (34.1% vs 11.8%; *p* < 0.01).

## Discussion

This study suggested that a tumor diameter of 3 cm could serve as a potential cutoff value for predicting early recurrence in patients with resectable PC, and a tumor diameter of ≥ 3 cm was identified as an independent risk factor for worse RFS and DSS for all patients undergoing pancreatectomy.

If early recurrence happens after highly invasive pancreatic resection, the surgery may reasonably be regarded as futile. Even with the adoption of neoadjuvant chemotherapy in recent years, tumors measuring ≥ 3 cm at the time of resection have little prognostic benefit from pancreatectomy and upfront surgery. In this study, large pancreatic cancers more frequently recurred locally than small pancreatic cancers, even after achievement of an R0 resection, suggesting that such tumors might not be truly “resectable”. It is extremely difficult to achieve a cure with surgery alone in such cases. More intensive perioperative therapy may overcome this limitation.

Tumor diameter is widely recognized as a critical prognostic factor in cancer, and the same holds true for pancreatic cancer. According to a nationwide Japanese survey published by Egawa et al.^6^ in 2012, the prognosis was clearly stratified by 1-cm increments. In the latest UICC staging system for pancreatic cancer, the T factor is determined based on tumor diameter and the presence or absence of arterial invasion.^7^

In this study, according to the distribution of Youden’s index plots presented, a tumor diameter of 3 cm was found to be a reasonable cutoff value for predicting an important prognostic parameter and early recurrence.^15,16^ It is particularly striking that the odds ratio for early recurrence in the large group was as high as 5.74 in the multivariable analysis. Of course, given the oncologic complexity of pancreatic cancer, it is unlikely that early recurrence is determined solely by tumor size, and further analyses incorporating biologic or radiologic markers will be necessary.

Many reports suggest that the 2-cm cutoff value for UICC staging of T1 best stratifies prognosis, which also has been confirmed in meta-analyses.^7,17–21^ However, these studies did not limit their analysis to resectable PC, including borderline PC with a smaller tumor diameter. The 3-cm cutoff value in this study is consistent with previous reports because we limited the subjects to those with resectable PC. The 2-cm cutoff is a truly important point for distinguishing prognosis, but it has been reported that in more than 70% of cases, pancreatic cancer is diagnosed with a cutoff of > 2 cm, making this criterion clinically less relevant.^17^ One important point to note in this discussion is that no comparison was made with non-surgical cases of tumors ≥ 3 cm in size. In the future, comparative studies including randomized trials with non-surgical cases are warranted.

Local recurrence, a common recurrence pattern in pancreatic cancer, is likely to be a failure of complete resection.^22,23^ To investigate why pancreatic resection for pancreatic cancer ≥ 3 cm in size is a poor prognostic factor, we examined the patterns of postoperative recurrence based on the pathologic R status. The results showed that in cases of R1 resection, both the large and small groups showed the same local recurrence rates. In contrast, with R0 resection, the local recurrence rate was only 11.8% in the small group, but it remained an unacceptably high 34.1% in the large group (*p* < 0.01). Despite achievement of R0 resection according to current pathologic standards, local recurrence still may occur, probably due to the inherent infiltrative nature of pancreatic cancer, microscopic residual disease beyond visible margins, and challenges in accurately diagnosing viable tumor cells on the resection margin.^24,25^

In the current study, microscopic lymphatic, vascular, and perineural invasions were more frequently observed in the large group (Table S1), indicating that the tumors in this group were biologically more aggressive. Although tumor margin measurements were not performed in this study, the larger tumor diameter in the large group suggests that the resection margins might inevitably become narrower.^26–28^ Although a larger surgical margin may enhance the curability of pancreatic cancer with extremely narrow margins, downsizing the tumor through chemotherapy may be more practical than extended resection.

In Japan, based on the clinical study results of the PREP-01 trial, the guidelines recommend two courses of GS therapy as neoadjuvant chemotherapy for resectable PC.^8,29^ In this study, GS was administered to 62 patients. As shown in Fig. 3, the prognosis for pancreatic cancer ≥ 3 cm in size after GS therapy was significantly worse than for tumors smaller than 3 cm, and comparable with that of tumors ≥ 3 cm in size treated with upfront surgery. A reduction in tumor size through neoadjuvant chemotherapy may be one of the key factors in achieving a favorable treatment outcome. However, the study also suggested that two courses of GS therapy may be insufficient in certain cases. In such cases, intensive chemotherapies such as gemcitabine/nab-paclitaxel or FOLFIRINOX, typically used for borderline and unresectable pancreatic cancer, may offer potential for effective tumor downsizing because previous studies have reported partial response rates of approximately 23%. However, because the response rate for GS neoadjuvant therapy has not been reported, a direct comparison between these regimens remains difficult.^30,31^ This reasoning is based on the fact that neoadjuvant chemotherapy for borderline PC leads to a higher rate of R0 resection.^32,33^ These findings warrant further investigation through prospective clinical studies.

This study had several limitations. First, it was a single-center retrospective observational study, which may have introduced biases in determining resectability and selecting surgical procedures. Second, the small sample, particularly of patients who underwent neoadjuvant chemotherapy, may have resulted in insufficient statistical power. Third, the study had limitations regarding tumor diameter measurements. In this study, pathologic evaluation was used to define tumor diameter. This decision was made because preoperative imaging measurements can vary depending on the examiner and imaging method, whereas pathologic tumor size reflects the true tumor size.

Additionally, some PC patients are contraindicated for contrast-enhanced CT due to contrast allergy or impaired renal function. However, it has been reported that assessment of tumor diameter by preoperative CT underestimates the pathologic tumor diameter by 20–25%.^17,34,35^ At our institution, the tumor diameter measured by preoperative CT was on the average 80.4% of the pathologic diameter compared with 84.1% when measured by EUS. In other words, using a 3-cm cutoff based on preoperative imaging tends to select tumors that actually are larger and associated with a poorer prognosis. Although this approach is acceptable in that it minimizes the risk of overlooking potentially curable cases, the development of more accurate diagnostic methods is warranted.

Finally, it is important to consider that withholding surgery for tumors larger than 3 cm at this stage could result in the loss of approximately 25% of potential long-term survivors. This highlights the need to establish improved treatment strategies through new approaches to multimodal therapy, including both pre- and postoperative chemotherapy.

## Conclusions

Early recurrence within 8 months after surgery for patients with resectable pancreatic cancer is associated with a poor prognosis, and a pathologic tumor size of ≥ 3 cm was found to be a strong predictive factor. Despite achievement of R0 resection, a high rate of local recurrence suggests that these cases may not represent truly “resectable” disease. This prognostic stratification is expected to contribute to the establishment of more effective multimodal treatment strategies in the future.

## Supplementary Information

Below is the link to the electronic supplementary material.

Supplementary file1 (DOCX 38 KB)

Supplementary file2 (TIF 104 KB)Comparison of the Kaplan-Meier curves between the futile surgery (FS) and no FS groups. Median OS was 1.2 years in the FS groupand 6.7 years in the no FS group, with a significant difference observed (p < 0.01).
