# Supplementary material for: Large Resectable Pancreatic Cancer is Associated with Futile Surgery: A Resectable But Not Curable Disease?
Source: Ann Surg Oncol. 2025 Oct 27;33(2):1595–604. doi: 10.1245/s10434-025-18511-2 (PMC12765747; doi:10.1245/s10434-025-18511-2)
Supplement: Supplementary file 1 — Supplementary file1 (DOCX 38 KB) [file 10434_2025_18511_MOESM1_ESM.docx]

**Supplementary Table 1. Pathological findings between Large and Small groups**

|  | Large group | Small group |  |
| --- | --- | --- | --- |
| Variables | n=206 | n=163 | p |
| Tumor diameter, cm | 4.1 (3.0–15.0) | 1.9 (0.1–2.9) | <0.01 |
| Tumor differentiation |  |  | 0.12 |
| Well, n (%) | 85 (41.3) | 85 (52.1) |  |
| Moderate, n (%) | 86 (41.7) | 60 (36.8) |  |
| Poor, n (%) | 19 (9.2) | 12 (7.4) |  |
| Others, n (%) | 16 (7.8) | 6 (3.7) |  |
| Microscopic lymphatic invasion, yes, n (%) | 132 (64.1) | 58 (35.6) | <0.01 |
| Microscopic venous invasion, yes, n (%) | 192 (93.2) | 131 (80.4) | <0.01 |
| Microscopic perineural invasion, yes, n (%) | 195 (94.7) | 119 (73.0) | <0.01 |
| Extrapancreatic invasion, yes, n (%) | 197 (95.6) | 117 (71.8) | <0.01 |
| Lymph nodes metastasis, yes, n (%) | 146 (70.9) | 71 (43.6) | <0.01 |
| R status, R1 resection, n (%) * | 42 (20.4) | 19 (11.7) | 0.03 |
| Pancreas cut margin, positive, n (%) | 18 (8.8) | 11 (6.7) | 0.46 |
| Bile duct cut margin, positive, n (%) | 0 (0.0) | 0 (0.0) | - |
| Dissected peripancreatic margin, positive, n (%) | 31 (15.0) | 10 (6.1) | <0.01 |

Data are presented as medians and ranges, unless otherwise indicated. P-values were calculated using the Mann–Whitney U test or chi-square test, as appropriate.

* These are duplicates.

**Supplementary Table 2. Univariable and multivariable Cox proportional hazards model analyses for DSS (n=369).**

|  |  | Univariable | | | Multivariable | | |
| --- | --- | --- | --- | --- | --- | --- | --- |
| Variables | n | HR | 95% CI | p | HR | 95% CI | p |
| Age, years, ≥75 | 140 | 1.06 | 0.79–1.41 | 0.69 |  |  |  |
| Tumor location, Head | 212 | 1.31 | 0.99–1.73 | 0.06 |  |  |  |
| Neoadjuvant chemotherapy, no | 300 | 1.18 | 0.78–1.77 | 0.44 |  |  |  |
| Tumor diameter, ≥3 cm | 206 | 2.65 | 1.97–3.56 | <0.01 | 2.15 | 1.58–2.93 | <0.01 |
| CA19-9, ≥500 U/mL | 57 | 2.22 | 1.59–3.10 | <0.01 | 1.54 | 1.09–2.18 | 0.02 |
| Tumor differentiation, without well | 200 | 1.41 | 1.07–1.86 | 0.02 | 1.16 | 0.87–1.54 | 0.32 |
| Lymphatic invasion, yes | 190 | 2.41 | 1.81–3.21 | <0.01 | 2.01 | 1.50–2.70 | <0.01 |
| Venous invasion, yes | 323 | 2.40 | 1.44–4.01 | <0.01 | 1.23 | 0.71–2.13 | 0.47 |
| Perineural invasion, yes | 314 | 2.68 | 1.63–4.41 | <0.01 | 1.15 | 0.64–2.09 | 0.63 |
| Lymph nodes metastasis, positive | 217 | 2.08 | 1.54–2.81 | <0.01 | 1.27 | 0.91–1.78 | 0.16 |
| R1 resection, yes | 61 | 2.04 | 1.46–2.85 | <0.01 | 1.84 | 1.32–2.59 | <0.01 |
| Adjuvant chemotherapy, no | 102 | 1.33 | 0.98–1.80 | 0.07 |  |  |  |

DSS, disease-specific survival; CA19-9, carbohydrate antigen 19-9; HR, hazard ratio; CI, confidence interval.
