# Supplementary figures and images for: Large Resectable Pancreatic Cancer is Associated with Futile Surgery: A Resectable But Not Curable Disease?
Source: Ann Surg Oncol. 2025 Oct 27;33(2):1595–604. doi: 10.1245/s10434-025-18511-2 (PMC12765747; doi:10.1245/s10434-025-18511-2)

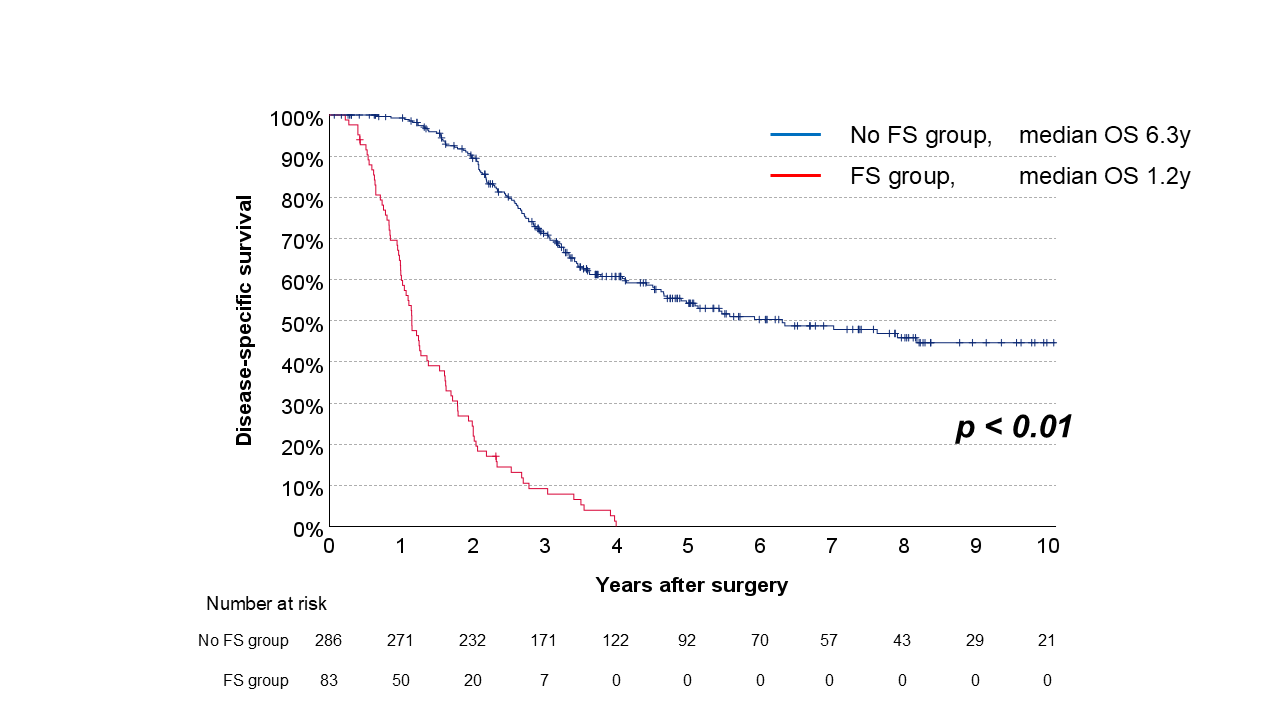

Supplement: Supplementary file 2 — Supplementary file2 (TIF 104 KB)Comparison of the Kaplan-Meier curves between the futile surgery (FS) and no FS groups. Median OS was 1.2 years in the FS groupand 6.7 years in the no FS group, with a significant difference observed (p < 0.01). [file 10434_2025_18511_MOESM2_ESM.tif]
